# Copaiba oleoresin as a substitute for sodium lasalocid in high‐concentrate diets for lambs: growth performance, ingestive behavior, and metabolic and ruminal parameters

**DOI:** 10.1002/jsfa.70894

**Published:** 2026-07-20

**Authors:** Jéssica Carvalho de Pantoja, Aylpy Renan Dutra Santos, Cristiane Rebouças Barbosa, Carla Giselly Souza, Tatiane Fernandes, Elaine Cristina Pacheco de Oliveira, Marcio Rodrigues de Souza, Fernando Miranda de Vargas Junior

**Affiliations:** ^1^ Department of Animal Science Faculty of Agricultural Sciences, Federal University of Grande Dourados Dourados Brazil; ^2^ School of Animal Science, Virginia Polytechnic Institute and State University Blacksburg VA USA; ^3^ Plant Biotechnology Laboratory Federal University of Western Pará Santarém Brazil; ^4^ Federal Institute of Mato Grosso do Sul Dourados Brazil

**Keywords:** active biocompounds, *β*‐caryophyllene, growth performance, ionophores

## Abstract

**BACKGROUND:**

The indiscriminate and excessive use of ionophore additives has been associated with the emergence of multi‐resistant pathogenic strains and the global spread of antibiotic resistance, raising serious public health concerns. Thus, plant biocompounds have been the focus of several studies aiming to replace ionophore additives in ruminant feeding without negatively impacting animal productivity. This study aimed to evaluate the effects of using copaiba oleoresin (CO) (*Copaifera reticulata*) as a substitute for sodium lasalocid (LAS) in high‐concentrate diets for feedlot‐finished lambs, analyzing intake, growth performance, digestibility, ingestive behavior, and metabolic and ruminal parameters.

**RESULTS:**

Sodium lasalocid treatment resulted in higher dry matter intake than the 1 mL CO dose (CO‐1.0) (*P* = 0.02) but did not differ significantly from the 0.5 mL CO dose (CO‐0.5). Similarly, LAS resulted in higher neutral detergent fiber rumination efficiency (32.17) then CO‐1.0 (24.60) (*P* < 0.01), but did not differ from CO‐0.5 (28.23). In the LAS group, ruminal pH (6.41) was slightly lower than the values observed in CO‐0.5 (6.77) and CO‐1.0 (6.84). The total concentration of short‐chain fatty acids and ammonia nitrogen decreased (*P* < 0.05) with the inclusion of CO‐0.5 and CO‐1.0.

**CONCLUSION:**

The use of copaiba oleoresin altered intake and ruminal parameters but did not affect growth performance; it therefore has the potential to be utilized in diets for feedlot‐finished lambs. © 2026 The Author(s). *Journal of the Science of Food and Agriculture* published by John Wiley & Sons Ltd on behalf of Society of Chemical Industry.

## INTRODUCTION

The evolutionary success of ruminants is largely due to the symbiotic relationship these animals maintain with the microorganisms that inhabit the rumen. Ruminants provide microorganisms with substrates and an environment that is ideal for the fermentation process, while the microorganisms provide ruminants with a variety of nutrients that are utilized by the animals for maintenance and production processes.^1^ However, during the fermentation process, some inefficiencies are observed, particularly the energy loss caused by enteric methane (CH_4_) production and the deamination of true protein.^2^ These processes reduce feed efficiency, impair the animals' growth performance, and increase environmental pollution.^3^

In this context, ionophore additives have been used widely in ruminant nutrition to reduce these inefficient processes and improve feed efficiency. The mechanism of action of ionophores mainly involves the reduction of the ruminal population of gram‐positive bacteria, decreasing the molar proportion of acetate and increasing the proportion of propionate.^4^ Gram‐positive bacteria are known for their direct role in protein degradation in the rumen and for contributing indirectly to CH_4_ production. In previous studies, ionophores reduced the degradation of true protein in the rumen,^5^ increased feed efficiency,^6^ and decreased enteric CH_4_ production.^7^

However, the excessive and indiscriminate use of ionophores may lead to the emergence of multi‐resistant pathogenic strains and the global spread of antibiotic resistance, resulting in a worldwide public health issue.^8^, ^9^, ^10^ In this regard, plant biocompounds have been the focus of several studies aiming to replace ionophore additives in ruminant feeding without negatively impacting animal productivity.^11^, ^12^

Copaiba oleoresin (CO) (*Copaifera reticulata*) is a natural product rich in active biocompounds, with sesquiterpenes making up the majority. The main sesquiterpene present in the composition of copaiba oleoresin is *β*‐caryophyllene, a biocompound that has antimicrobial properties against gram‐positive bacteria.^13^, ^14^, ^15^ Copaiba oleoresin has been evaluated as a ruminant diet additive because its diverse biocompounds provide potent antimicrobial effects with a lower risk of resistance development.^12^, ^16^, ^17^

However, the *in vivo* studies that used CO were conducted with grazing animals^18^ or in situations where the diet contained a higher proportion of roughage (530 g kg^−1^).^16^ The literature has documented that the use of high‐concentrate diets can enhance the action of active biocompounds in the rumen.^3^ In light of this, the aim of the current study was to evaluate the effects of using copaiba oleoresin as a substitute for LAS in high‐concentrate diets for feedlot‐finished lambs, analyzing feed intake, growth performance, digestibility, ingestive behavior, and metabolic and ruminal parameters.

## MATERIALS AND METHODS

### Location, procedures, and obtaining CO

The experiment was conducted at the Sheep Farming Sector of the Faculty of Agricultural Sciences of the Federal University of Grande Dourados (UFGD), located in the municipality of Dourados, Mato Grosso do Sul, Brazil (22° 14′ S, 54° 49′ W; altitude 450 m). According to the Köppen classification, the region's climate is of the Cwa type (humid mesothermal), with a rainy summer and dry winter. During the experimental period, the average temperature was 27.09 °C. The UFGD ethics committee on animal use approved the execution of this study under protocol no. 32/2020.

The CO used in this study was provided by the Plant Biotechnology Laboratory of the Federal University of Western Pará (UFOPA). The CO was collected in the Tapajós National Forest during the rainy season and was subsequently subjected to phytochemical analyses to determine its active biocompounds. The characteristics and content of active biocompounds were determined using gas chromatography coupled with mass spectrometry (GC–MS). The constituents were identified based on their retention indices, comparing them with standards published in the literature.^19^

### Animals, treatments, and management

A total of 24 male Pantaneiro lambs, with an average initial weight of 21 ± 3.9 kg and an average age of 152 days, were assigned to a randomized block design with three treatments and eight replicates. The animals' weight was the factor used for blocking. The experimental period lasted 56 days, with the first 14 days allocated for adaptation to the facilities, environment, and diet, while the subsequent 42 days were subdivided into three 14‐day periods for data collection. The lambs were individually housed in concrete pens (1.5 m × 1.5 m), with floors covered with rice straw. Each pen was equipped with an automatic waterer and a feeder.

The basal diet (Table 1) was formulated based on the nutritional requirements prescribed by the US National Research Council^20^ for lambs with a weight gain of 300 g day^−1^, assuming a dry matter intake (DMI) of 35 g kg^−1^ of body weight (BW). Thus, the diet contained 200 g kg^−1^ roughage (oat hay) and 800 g kg^−1^ concentrate (ground corn, soybean meal, a mineral mix specific for sheep, and calcium carbonate). The diet was offered to the animals daily in two distinct feedings, at 07:00 a.m. and 12:00 p.m. Feed allowance was adjusted every 3 days, based on daily leftovers to target a refusal margin of 50 g kg^−1^.

**Table 1:** Chemical and centesimal composition of the experimental diets

Ingredients	Quantity (g kg^−1^)
Hay	200.0
Corn	632.0
Soybean meal	152.0
Mineral	8.0
Calcium carbonate	8.0
Composition	
Dry matter (g kg^−1^ of diet)	895.1
Organic matter (g kg^−1^ of DM)	918.2
Crude protein (g kg^−1^ of DM)	210.2
Ether extract (g kg^−1^ of DM)	35.0
Neutral detergent fiber (g kg^−1^ of DM)	247.0
Acid detergent fiber (g kg^−1^ of DM)	137.8
Total carbohydrates (g kg^−1^ of DM)	673.0
Non‐fibrous carbohydrates (g kg^−1^ of DM)	426.0
Total digestible nutrients (g kg^−1^)	877.2

*Note:* DM refers to Dry Matter.

The treatments used in this study were as follows: LAS – 22.5 mg kg^−1^ of LAS (Taurotec‐Zoetis, Campinas‐SP, Brazil), containing 150 g kg^−1^ sodium lasalocid in the diet; CO‐0.5 – administration of 0.5 mL per animal per day of CO; and CO‐1.0 – administration of 1.0 mL per animal per day of CO. The doses of CO were defined according to a study by Moura *et al*.,^16^ with a modification in the administration method by which the doses were provided orally with the aid of a graduated syringe, ensuring daily CO intake regardless of the animal's consumption, thus minimizing potential losses of volatile compounds. The LAS was added to the diet according to the manufacturer's recommendation at a ratio of 0.150 g kg^−1^ of diet, providing sodium lasalocid at a dose of 22.5 mg kg^−1^ of diet. The justification for not including a negative control (without additive) was the high incidence of *Eimeria* spp. at the study site, which is associated with high mortality rates.

### Feed intake, productive performance, and digestibility

Daily, before offering the diet, the leftovers from the previous day were collected from the feeders and weighed to determine DMI and nutrient intake. At the end of each data collection period, samples of ingredients, diet, and leftovers from each animal were collected, identified, and stored in plastic bags, then frozen at −20 °C for later chemical composition analysis. Lambs were also weighed after a 16 h fasting period at the end of each experimental period to monitor performance. The final body weight (FBW) presented in Table 2 corresponds to the BW recorded after a 16 h fasting period on the last day of the experiment and was used to calculate average daily gain (ADG) and feed efficiency (FE). The FE was obtained from the ratio between the ADG and the daily DMI.

**Table 2:** Intake, growth performance, and digestibility coefficients of diets based on the use of sodium lasalocid or different doses of copaiba oleoresin

Items	Treatments^a^			SEM^b^	*P*
	LAS	CO‐0.5	CO‐1.0		
Intake (g day^−1^)					
Dry matter	830.18^a^	807.69^a^	728.79^b^	41.548	0.02
Organic matter	793.08^a^	771.32^a^	696.96^b^	39.448	0.02
Crude protein	172.33^a^	166.54^a^	150.47^b^	8.740	0.01
Ether extract	17.19	16.63	15.02	1.280	0.38
Neutral detergent fiber	192.06^a^	175.22^ab^	155.75^b^	10.840	0.03
Acid detergent fiber	65.01	68.41	65.76	4.067	0.75
Total carbohydrates	603.98^a^	602.10^a^	529.14^b^	32.320	<0.01
Non‐fibrous carbohydrates	409.90	411.92	376.29	10.46	0.17
Growth performance					
Initial body weight (kg)	21.21	20.06	21.07	‐	‐
Final body weight (kg)	31.15	29.33	30.36	1.787	0.40
Average daily gain (g)	236.67	220.71	221.19	13.73	0.69
Feed efficiency	0.28	0.27	0.31	0.021	0.26
Digestibility (g kg^−1^)					
Dry matter	791.3	823.0	863.9	27.25	0.22
Organic matter	797.8	827.8	867.3	26.53	0.23
Crude protein	833.6	847.2	877.2	22.26	0.40
Ether extract	811.8	850.6	864.1	31.29	0.43
Neutral detergent fiber	590.7	659.0	627.3	60.32	0.73
Acid detergent fiber	400.8	667.6	681.8	83.23	0.07
Total carbohydrates	827.3	864.2	843.0	12.13	0.45
Non‐fibrous carbohydrates	749.0	815.5	766.1	17.22	0.23
Total digestible nutrients	758.4	792.3	831.2	23.71	0.15

^a^ LAS, 22.5 mg kg^−1^ of LAS in the diet; CO‐0.5, 0.5 mL of CO per animal per day; CO‐1.0, 1.0 mL of CO per animal per day.

^b^ SEM, standard error of the mean.

*Note:* Means followed by different superscripts (^a–b^) within a row differ significantly according to Tukey's test (*P* < 0.05).

To evaluate nutrient digestibility, total feces were collected over 24 h on 12 lambs (four random blocks) using individual fecal collection bags. The collection was carried out on days 9, 10, and 11 of each period. After collection, the feces were weighed, and a 300 g kg^−1^ subsample from each animal was identified, stored in individual plastic bags, and frozen at −20 °C for later chemical analysis.

After the experimental period, samples of ingredients, diet, leftovers, and feces were thawed, pre‐dried in a forced air circulation oven at 65 °C for 72 h, and then ground in a Wiley‐type knife mill equipped with 1 mm mesh sieves. From these ground samples, dry matter (DM, ID 934.01), ash (ID 934.05), crude protein (CP, ID 981.10), and ether extract (EE, ID 920.39) content was determined according to the methodologies proposed by AOAC.^21^ The organic matter (OM) content was determined by the difference between dry matter and ash content. Neutral detergent fiber (NDF) and acid detergent fiber (ADF) content were obtained according to the method proposed by Van Soest *et al*.^22^ with adaptations.^23^ Total carbohydrates (TC) were calculated based on the methodology described by Sniffen *et al*.^24^: using the formula

*TC* (g kg^−1^) = 100 − (*CP* + *EE* + ash)

Non‐fiber carbohydrates (NFC) were determined using the equation:

*NFC* (g kg^−1^) = *TC* – *NDF*

The total digestible nutrients (TDN) in the forage were obtained in proportion to the forage‐to‐concentrate ratio offered. The TDN of the forage was calculated using the equation

*TDN* g kg^−1^ = 83.79 – (0.4171 × *NDF*)

as described by Cappelle *et al*.,^25^ and the estimated TDN for the concentrate was calculated using the equation

*TDN* g kg^−1^ = (88.9 – (0.779 × *ADF*))

as described by Patterson *et al*.^26^

The digestibility coefficient was obtained using the equation proposed by Silva and Leão:^27^

digestibility (g kg^−1^) = [(nutrient ingested – nutrient in feces) / nutrient ingested] × 100

The TDN intake was determined from the equation described by Weiss *et al*.:^28^

*TDN* (g kg^−1^) = *CPd* + (*EEd* × 2.25) + *NFCd* + *NDFd*

where *CPd* = digestible crude protein, *EEd* = digestible ether extract, *NFCd* = digestible non‐fibrous carbohydrates, and *NDFd* = digestible neutral detergent fiber.

### Ingestive behavior

Ingestive behavior was investigated by individual visual observations beginning at 07:00 a.m. on the eighth day of each period, at 5 min intervals over a continuous 24 h period.^29^ Visual observations were made by trained observers, who recorded the time each animal spent feeding, ruminating, drinking water, and idling. Total chewing time was obtained by summing the time spent on ingestion and rumination.

The feeding and rumination efficiencies of DM and NDF were calculated according to the model proposed by Polli *et al*.:^30^

FE_DM_ = DMI FT^−1^;

RE_DM_ = DMI RT^−1^

RE_NDF_ = NDFI RT^−1^

where FE is feeding efficiency, FT is feeding time, NDFI is neutral detergent fiber intake, RE is rumination efficiency, and RT is rumination time. Feeding and rumination times were divided by 60 to express efficiencies in hours. The DMI and NDFI values used in these calculations corresponded to the days of the ingestive behavior evaluations.

On the same day, the number of rumination chews was counted during four different periods (10:00 a.m.–12:00 p.m.; 16:00 p.m.–18:00 p.m.; 22:00–00:00 a.m.; and 04:00–06:00 a.m.). In each period, three ruminal boli were observed per animal, totaling 12 boli per animal per day. The number of rumination chews and the time spent (in seconds) ruminating each bolus were recorded using a digital stopwatch. The number of boli ruminated, chewing time per bolus, number of chews per bolus, and total daily chews were determined as described by Polli *et al*.^30^

### Metabolic and ruminal parameters

On the last day of each period, 4 h after the first daily feeding, blood samples were collected from the animals by jugular vein puncture using Vacutainer tubes for the determination of plasma urea nitrogen (PUN), glucose, cholesterol, aspartate aminotransferase (AST), and alanine aminotransferase (ALT). The samples were centrifuged at 3000 × *g* for 20 min at 4 °C to obtain plasma, which was stored in Eppendorf‐type tubes and frozen at −20 °C. Later, the plasma samples were thawed at room temperature and analyzed according to the methods described by Campos *et al*.,^31^ using a semi‐automatic analyzer and commercial kits from Bioclin –(Belo Horizonte, Brazil).

On the last day of the feedlot period (day 42), the lambs were subjected to a 16 h solid feed fasting period and subsequently slaughtered. The slaughter was performed following the Regulations of the Industrial and Sanitary Inspection of Animal Origin Products (RIISPOA).^32^ The animals were stunned with an electric shock before bleeding. Data related to carcass characteristics and meat quality of these animals can be found in Santos *et al*.^12^

Urine was collected directly from the bladder during slaughter. An aliquot of 10 mL of urine was diluted in 40 mL of 0.036(eq/L)  sulfuric acid for the determination of urinary concentrations of creatinine, urea, and allantoin, according to the protocol standardized by Chen *et al*.^33^ Daily urine output was estimated from spot urine creatinine concentration, using a fixed species‐specific creatinine excretion rate and the metabolic weight estimated from bodyweight. Subsequently, urea and allantoin output were calculated based on spot concentration and daily urine output as described by Chen *et al*.^33^

To determine pH and the concentrations of short‐chain fatty acids (SCFAs) and ammonia nitrogen (N‐NH_3_) in the ruminal fluid, a sample of ruminal content was collected during slaughter.^34^ The pH was measured immediately after collection using a digital pH meter. Two subsamples of the filtered content were strained through gauze, stored, and frozen for subsequent SCFA and N‐NH_3_ analyses.

For the determination of SCFAs, the ruminal fluid sample was centrifuged at 7000 × *g* for 15 min at 4 °C, and the supernatant was filtered through polytetrafluoroethylene (PTFE) membrane filters (33 mm, 0.22 μm) and stored at −20 °C for subsequent analysis. Volatile fatty acids were determined using high‐performance liquid chromatography (HPLC) (Shimadzu, Prominence model, Kyoto, Japan), equipped with a UV detector (SPD‐20) programmed to operate at a wavelength of 210 nm. The system was equipped with an Aminex HPX‐87H column with dimensions of 300 × 7.8 mm and a particle diameter of 9 μm, operated at 30 °C. The mobile phase consisted of 5 mmol L⁻¹ H_2_SO_4_ in isocratic mode for 37 min. The N‐NH_3_ concentration was determined using the Kjeldahl method, as proposed by Detmann *et al*.^35^

### Statistical analyses

The data were checked for assumptions (normality and homoscedasticity) and then analyzed using the PROC MIXED procedure of SAS (SAS Institute Inc., Cary, NC, USA). Means were compared using the Tukey test, with *P* < 0.05 indicating a significant difference. The statistical model used was: *Y*_ij_ = *μ* + *β*_j_ + *T*_i_ + *ϵ*_ij_, where: *Y*_ij_ = observed value; *μ* = overall mean of the response variable; *β*j = block effect (j: 1–8); Ti = treatment effect (LAS, CO‐0.5, and CO‐1.0), and *ϵ*_ij_ is the experimental error associated with the observed value *ij*. For observations of digestibility, behavior, and plasma metabolite that were conducted at each period, time and the interaction of treatment and time were included in the model. Time was therefore used as a repeated effect, and a first‐order autoregressive (AR (1)) variance structure were adopted. The AR (1) structure was selected over compound symmetry and unstructured forms based on the Akaike information criterion (AIC).

## RESULTS

Among the active biocompounds identified in CO, *β*‐caryophyllene showed the highest concentration at 223.0 g kg^−1^, followed by *α*‐bergamotene, *γ*‐macrocarpene, and *β*‐selinene, which accounted for 208.0 g kg^−1^, 151.0 g kg^−1^, and 130.0 g kg^−1^ of CO, respectively (Table 3).

**Table 3:** Chemical composition and active biocompounds of copaiba oleoresin

Analytical indices	Copaiba oleoresin
Acidity (mg g^−1^)	86.10 ± 2.02
Saponification (mg g^−1^)	99.15 ± 0.89
Refraction	1.50 ± 0.02
Density (g mL^−1^)	0.98 ± 0.003
Viscosity (Pa‐s)	0.28 ± 0.01
Chemical components (g kg^−1^)	
*β*‐Caryophyllene (C_15_H_24_)	223.2
*α*‐Bergamotene (C_15_H_24_)	208.3
*γ*‐Macrocarpene	151.4
*β*‐Selinene (C_15_H_24_)	130.6
*α*‐Selinene	64.0
*α*‐Humulene	39.5
*β*‐Elemene	30.4
Caryophyllene oxide (C_15_H_24_O)	22.8
*β*‐Sesquifelandrene	19.7
*α*‐Bisabolene	19.2
Amorpha‐4,7(11)‐diene	13.1
Junenol	12.1
*β*‐Farnesene	11.4
Pogostol	11.4
*δ*‐Cadinene (C_15_H_24_)	10.8
*α*‐Aromadendrene (C_15_H_24_)	3.1
*γ*‐Curcumene	2.9
*α*‐Copaene (C_15_H_24_)	2.9
*β*‐Curcumene	2.5
Caryophyllene<14‐hydroxy‐9‐epi‐(E)‐>	2.4
*γ*‐Bisabolene	1.3
*α*‐Bisabolol	0.90
*δ*‐Elemene	0.50

Table 2 presents data on intake, growth performance, and digestibility. Sodium lasalocid resulted in higher DMI than CO‐1.0 (*P* = 0.02), with no difference from CO‐0.5. Similarly, the intakes of OM, CP, and TDN followed the same pattern (*P* < 0.05). The use of CO‐1.0 reduced NDF intake (*P* = 0.03) compared with LAS, but did not differ from CO‐0.5, which, in turn, yielded similar values to LAS. The growth performance of the lambs did not differ significantly (*P* > 0.05) among treatments, as the average values obtained for FBW, ADG, and FE were similar. The digestibility coefficients did not differ statistically (*P* > 0.05) among the evaluated treatments.

Table 4 shows that the treatments influenced the ingestive behavior of the animals. Sodium lasalocid resulted in higher NDF rumination efficiency (32.17) than CO‐1.0 (24.60) (*P* < 0.01), but did not differ from CO‐0.5 (28.23). Regarding the number of ruminating chews per minute, CO‐0.5 and CO‐1.0 resulted in higher values than LAS. The other parameters related to ingestive behavior were similar statistically among treatments (*P* > 0.05). However, the feeding time with CO‐0.5 was 35% higher and with CO‐1.0 it was 19% higher than with the LAS treatment.

**Table 4:** Ingestive behavior of lambs fed diets containing sodium lasalocid or doses of copaiba oleoresin

Item	Treatments^a^			SEM^b^	*P*
	LAS	CO‐0.5	CO‐1.0		
Feeding time (min day^−1^)	155.00	208.96	185.00	16.991	0.10
Rumination time (min day^−1^)	371.67	386.46	387.08	16.625	0.72
Chewing time (min day^−1^)	526.67	595.42	572.08	24.373	0.14
Water intake time (min day^−1^)	8.75	7.08	6.88	1.809	0.73
Idling time (min day^−1^)	904.17	836.25	860.21	25.166	0.17
Feeding efficiency (g DM h^−1^)	369.49	269.89	257.65	41.879	0.15
Rumination efficiency (g DM h^−1^)	136.87	124.11	115.92	7.753	0.17
Rumination efficiency (g NDF h^−1^)	32.17^a^	28.23^ab^	24.60^b^	1.980	<0.01
Time of chews per bolus (sec)	47.20	46.61	44.46	2.062	0.62
Number of chews per bolus	80.08	87.05	82.75	3.653	0.39
Number of chews per min	102.27^b^	112.24^a^	111.67^a^	1.636	<0.01
Number of ruminated boli per day	485.28	504.64	539.21	36.552	0.58
Number of chews per day	38 399	43 433	43 274	2182.60	0.42

^a^ LAS, 22.5 mg kg^−1^ of LAS in the diet; CO‐0.5, 0.5 mL of CO animal^−1^ day^−1^; CO‐1.0, 1.0 mL of CO animal^−1^ day^−1^.

^b^ SEM, standard error of the mean.

*Note:* Means followed by different superscripts (^a–b^) within a row differ significantly according to Tukey's test (*P* < 0.05).

Abbreviations: DM, Dry Matter; NDF, Neutral Detergent Fiber.

Table 5 reports the data regarding ruminal, blood, and urinary parameters. With the LAS treatment, the ruminal pH value (6.41) was slightly lower than the values observed in CO‐0.5 (6.77) and CO‐1.0 (6.84). Total volatile fatty acids (VFA) and N‐NH₃ concentrations decreased (*P* < 0.05) with the provision of CO‐0.5 and CO‐1.0. Regarding blood parameters, only AST concentration was influenced by the treatments, with LAS and CO‐0.5 resulting in higher values (*P* < 0.05) than those observed in CO‐1.0. Urinary parameters were not influenced (*P* > 0.05) by the studied treatments.

**Table 5:** Ruminal and metabolic parameters of lambs fed diets containing sodium lasalocid or copaiba oleoresin doses

Items	Treatments^a^			SEM^b^	*P*
	LAS	CO‐0.5	CO‐1.0		
Ruminal					
pH	6.41^b^	6.77^a^	6.84^a^	0.123	0.02
Acetate (g kg^−1^)	718.7	698.8	658.3	53.56	0.48
Propionate (g kg^−1^)	224.6	231.9	302.4	42.03	0.29
Butyrate (g kg^−1^)	56.7	69.3	39.3	14.39	0.14
Short‐chain fatty acid totals (mM)	96.13^a^	50.97^b^	61.81^b^	10.949	0.01
Acetate: propionate ratio	3.20	3.01	2.20	0.729	0.51
N‐NH_3_ (mg dL^−1^)	1.94^a^	0.96^b^	1.13^b^	0.329	0.04
Blood					
Glucose (mg dL^−1^)	59.25	71.88	76.29	5.135	0.06
Cholesterol total (mg dL^−1^)	72.62	65.88	49.25	7.255	0.09
Urea nitrogen (mg dL^−1^)	55.75	50.88	50.71	5.405	0.73
ALT (mg dL^−1^)	18.88	18.75	16.25	2.393	0.69
AST (mg dL^−1^)	115.88^a^	116.63^a^	80.75^b^	10.219	0.03
Urinary					
Urea (mg day^−1^)	3292.43	3648.66	3853.97	648.370	0.73
Allantoin (mg day^−1^)	1421.10	845.95	1237.40	733.630	0.77

^a^ LAS, 22.5 mg kg^−1^ of LAS in the diet; CO‐0.5, 0.5 mL of CO animal^−1^ day^−1^; CO‐1.0, 1.0 mL of CO animal^−1^ day^−1^.

^b^ SEM, standard error of the mean.

*Note:* Means followed by different superscripts (^a–b^) within a row differ significantly according to Tukey's test (*P* < 0.05).

## DISCUSSION

Based on the active biocompounds present in CO, *β*‐caryophyllene was expected to exhibit the greatest biological activity. In other studies, a higher concentration of *β*‐caryophyllene in CO has also been demonstrated.^36^, ^37^ *β*‐Caryophyllene has shown negative effects against gram‐positive bacterial strains,^14^, ^15^, ^38^ supporting the use of CO as a potential ruminal modulating additive. In the present study, the use of CO‐1.0 allowed for a greater supply of this active compound to the animals.

The reduction in DM and nutrient intake in CO‐1.0 demonstrates that the effects of biocompounds on intake in ruminants are dose dependent, being more effective when high doses are administered.^12^, ^39^ However, the literature on the effects of CO as a source of biocompounds on intake in ruminant animals still shows contradictory outcomes. Similar results to those of the present study were obtained by Teobaldo *et al*.,^40^ who supplemented pasture‐fed cattle with a blend containing CO in its composition. The authors observed that the effects of the blend are dose‐dependent, as the highest dose of this additive (300 mg kg^−1^ DM consumed) resulted in a decrease in DMI. However, Moura *et al*.^16^ reported that the inclusion of different doses (0.5, 1.0, or 1.5 g kg^−1^ of DM) of CO in the diet of confined lambs did not affect DMI. In a study conducted with rats, Pulcinelli *et al*.^41^ observed that the administration of CO reduced food intake, with this result attributed to *β*‐caryophyllene. The mechanism of action of this sesquiterpene on food intake has not yet been elucidated in the literature, limiting a more detailed explanation in the present study.

Even with the lower DM and nutrient intake in CO‐1.0 compared with LAS, animal growth performance did not differ among treatments. This result may be associated with the digestibility values, which, although not statistically significant, were numerically higher in CO‐1.0. The diet was high in concentrate, so this greater digestibility may have altered the ruminal fermentation pattern, resulting in increased propionate production despite the reduction in total SCFA concentration. The positive effects of active biocompounds on DM digestibility have been reported in previous studies.^42^, ^43^ In this context, it is hypothesized that the active biocompounds present in CO promoted ruminal propionate production. As propionate is the primary precursor of glucose in ruminants, although no statistically significant differences were detected in the present study, absolute serum glucose concentrations increased with CO supplementation, particularly in the CO‐1.0 treatment, possibly as a consequence of the greater propionate production. It is also hypothesized that the greater glucose supply provided by CO more efficiently met the animals' energy requirements, which may have contributed to the reduction in DM intake, resulting in numerically greater feed efficiency, particularly in CO‐1.0.

The concentration of N‐NH_3_ in CO‐1.0 was also lower than LAS, suggesting lower ruminal protein degradation and a greater amino acid supply to the intestine, resulting in higher absorption of high‐quality protein. In a meta‐analysis study with beef cattle, Orzuna‐Orzuna *et al*.^44^ observed that the inclusion of essential oils, rich in terpenes, caused a reduction in the ruminal concentration of N‐NH_3_. The reduction in N‐NH_3_ was due to the negative effects of the biocompounds in CO on gram‐positive bacteria, which are primarily responsible for the degradation of true protein in the rumen.^45^ The terpenes present in CO can also interact with food proteins to form complexes, preventing degradation by ruminal bacteria.^46^ According to Hart *et al*.,^47^ these biocompounds may inhibit ammonia‐overproducing bacteria, which are directly involved in the deamination of amino acids.

In the present study, the higher RE_NDF_ of the lambs fed with LAS occurred due to the higher consumption of NDF. Moura *et al*.^16^ reported that the use of 0.5 g kg^−1^ of DM of copaiba oleoresin increased the time spent on lambs’ feeding activities but did not change the efficiencies of DM and NDF intake. Dias Junior *et al*.^48^ did not observe changes in the ingestive behavior of lambs receiving essential oil of *Arnica montana* in the diet. In the present study, although no statistical differences were observed, the feeding time in LAS was shorter than the other treatments, even though this additive promoted higher DMI, particularly when compared with CO‐1.0. Hypothetically, the provision of CO may have reduced the passage rate, increasing the retention time of feed in the rumen and, consequently, requiring a greater number of feeding events. This reduced passage rate may have contributed to the higher, albeit numerical, values of DM digestibility observed with CO supplementation. However, the mechanisms by which CO could influence passage rate still require further investigation.

Diets with high concentrate content, like the one in the present study, tend to provide less stimulation for mastication, which can make the ruminal environment more acidic due to the lower pH.^3^ Despite the high concentrate content in the basal diet of the present study, the use of LAS, CO‐0.5, and CO‐1.0 maintained the pH at levels considered ideal for the fermentation process. The higher pH values in CO‐0.5 and CO‐1.0 may be related to the effects of the active biocompounds present in CO on the ruminal fermentation pattern, as reflected by the lower concentration of SCFA and N‐NH_3_. The lower SCFA concentration likely contributed to the higher ruminal pH values observed in the CO treatments due to the lower production of acids during fermentation. These results suggest that, although ruminal SCFA concentration was reduced, the animals' energy supply was not compromised, possibly due to the increased availability of glucose, as previously described. However, the mechanisms responsible for this response still need to be elucidated.

Khateri *et al*.^49^ did not observe changes in AST concentration in sheep fed different doses of an essential oil blend. The lower blood concentration of the AST enzyme due to CO‐1.0 clearly shows that the use of CO may have beneficial effects on cellular integrity, indicating a lower risk of liver or muscle damage. An evaluation of the liver tissue lesion score could corroborate these findings; however, it was not included in this study.

Although the use of different doses of copaiba oleoresin promoted a decrease in the total production of SCFAs, no negative impacts on growth performance were observed. This shows that copaiba oleoresin has the potential to be used as an additive in ruminant diets. The administration of copaiba oleoresin at a dose of 0.5 mL lamb^−1^ day^−1^ is already sufficient to replace the use of the ionophore LAS in high‐concentrate diets for feedlot‐finished lambs. It is recommended that more research should be carried out with different doses and other forms of administration to the animals.

## FUNDING INFORMATION

This work was supported by Coordination for the Improvement of Higher Education Personnel (CAPES) (Scholarship and Postgraduate Support Program (PROAP); Graduate Development Program (PDPG) – Strategic Consolidation 3–4, Process no. 88881.745570/2022‐01); the Foundation for the Support of Education, Science, and Technology Development in the State of Mato Grosso do Sul (FUNDECT) (Midwest Graduate Development Program (PDPG Centro‐Oeste), Process no. 83/035.027/2024, FUNDECT Grant Agreement no. 274/2024, Integrated Financial Administration and Control System (SIAFIC) no. 1481; State Graduate Development Program (PDPG Estados), FUNDECT Grant Agreement no. 133/2023, State Financial Administration System (SIAFEM) no. 33108); and the National Council for Scientific and Technological Development (CNPQ) (Research Productivity Fellowship (PQ Researcher)).

## CONFLICT OF INTEREST

The authors declare no conflicts of interest.

## ETHICAL APPROVAL

The UFGD ethics committee on animal use approved the execution of this study under protocol no. 32/2020.

## Data Availability

The data that support the findings of this study are available from the corresponding author upon reasonable request.
